# Validation of ToucHb, a non-invasive haemoglobin estimation: Effective for normal ranges, needs improvement for anaemia detection

**DOI:** 10.1371/journal.pgph.0001541

**Published:** 2024-03-12

**Authors:** Yogish Channa Basappa, Sumanth Mallikarjuna Majgi, Shashidhar Byrappa Shashidhar, Prashanth Nuggehalli Srinivas

**Affiliations:** 1 Institute of Public Health Bengaluru, Bengaluru, Karnataka, India; 2 Department of Community Medicine, Mysore Medical College and Research Institute, Mysore, Karnataka, India; 3 Department of Pathology, Mysore Medical College and Research Institute, Mysore, Karnataka, India; McMaster University, CANADA

## Abstract

Non-invasive methods for haemoglobin estimation hold enormous potential for early detection and treatment of anaemia, especially in limited resource settings. We sought to validate the diagnostic accuracy of ToucHb, a non-invasive haemoglobin estimation device available in the Indian market. We prospectively evaluated the diagnostic performance of the ToucHb device using the Automated complete blood count (CBC) method as the gold standard. Persons referred for haemoglobin estimation to the central laboratory of the government medical college hospital in Mysore, southern India were included in the study. Out Of 140 people approached, 127 gave consent; 65% (n = 82) were female with median age of 37 (IQR 28–45). ToucHB reported median haemoglobin value of 14 g/dL compared to 13.3 g/dL for CBC. Within 1 g/dL and 2 g/dL of CBC, 55.2% (70/127) and 74% (94/127) of ToucHb haemoglobin observations fell, respectively. The Bland-Altman plot showed a mean difference of 3 g/dL in haemoglobin between ToucHb and CBC among those with anaemia. The ToucHb device showed 22.2% sensitivity and 94.5% specificity for anaemia detection. In rural resource-limited settings, point of care non-invasive devices such as ToucHb can improve access and acceptance for anaemia screening. However, ToucHb has showed low sensitivity for anaemia detection and low accuracy at lower haemoglobin values. The utility of the instrument is especially limited in detecting anaemia, while it can estimate haemoglobin accurately among those with haemoglobin is in the normal range. Based on these findings, ToucHb and devices that work on the core technology deployed in ToucHb may be better suited to monitor known haemoglobin level rather than in anaemia screening or detection in primary/ secondary care and community settings.

## Introduction

In 2019, Iron deficiency anaemia was the second leading cause of healthy years of life lost due to disability among adolescents (aged 10–18) worldwide, and one of the top five causes overall years of life lost [1–3]. Anaemia is a major public health challenge in India, with high prevalence in pregnancy (52.2%) and children (6–59 months) (67%) [4]. The risk factors for anaemia are closely linked to socio-economic conditions including poverty, food insecurity and access to primary health care services especially in rural areas [5, 6]. Anaemia has a significant impact on child and overall human development, and reducing its burden is essential for achieving United Nations sustainable development goals [7].

The most reliable indicator for detection, management, and follow-up of anaemia at population level is blood haemoglobin concentration [8]. The World Health Organization (WHO) recommends the direct cyanmethaemoglobin and the HemoCue to estimate haemoglobin concentration at population level in large surveys in remote settings for high precision. However, this method requires skilled technicians for its operation [9]. Wherever, this is not feasible, WHO recommends the use of haemoglobin colour scale as a simple and inexpensive procedure [10, 11]. In most government primary healthcare centres in India, Sahli’s haemoglobinometer is used. This method has been shown to be marginally better than the haemoglobin colour scale but with shortcomings [12]. All these methods are invasive, requiring the collection of capillary or venous blood. This means that they require a trained person to follow the steps of finger prick, which can be a painful and uncomfortable [8, 9].

Non-invasive methods for anaemia detection have the potential to revolutionize early detection in limited resource settings. There have been multiple efforts in the last decade to develop innovative non-invasive methods for haemoglobin detection [13–16]. These methods are painless, quick, easy to use, and accessible to people in remote or underserved areas. They are also more likely to be tolerated by patients and reduce the risk of infection compared to invasive methods [17, 18]. As part of the Towards Health Equity and Transformative Action on tribal health (THETA) project, we sought to assess health inequities in remote and forested areas [19]. We explored the possibility of using non-invasive haemoglobin estimation technology for its operational feasibility and ethical benefits. In 2018–19, we chose the ToucHb device, which was the most easily available non-invasive device in India at the time. The device uses a smartphone camera with an accessory to capture an image of the conjunctiva and uses reflectance photometry to estimate haemoglobin levels in the blood [20].

An earlier evaluation of the device reported a sensitivity of 73% and specificity of 51% in a tertiary care hospital [21]. Before deploying ToucHb in multiple remote locations for survey in community settings, we sought to test and validate its results against a gold standard. Given the potential for using this device in screening the community and use in public health programs, there is a need to assess its accuracy for detecting haemoglobin in comparison to standardised method (cyan-methaemoglobin method of haemoglobin estimation using cyanide-free sodium lauryl sulphate reagent).

## Method

A prospective diagnostic evaluation study was designed and implemented at the central laboratory of Krishna Rajendra hospital attached to the Mysore Medical College and Research Institute, Mysore, India. The study was conducted between 31 December 2018 to 29 March 2019.

### Sample size

The Bland-Altman method is a common approach to evaluate the agreement between two measurement techniques [22, 23]. We estimated that a sample size of 124 would be sufficient to compare the ToucHB and Automated complete blood count (CBC) methods using the Bland-Altman method with noncentral t-distribution, with a mean difference of 0.3, a standard deviation of 0.5, and a power of 80%. By accounting 10% non-response rate, total sample size was 138. The R script for the sample size calculation is available in S1 Code [24].

### Sampling strategy

Purposive sampling strategy was used to conduct the study. Adults (age >18 years) who were referred by the physician to the central laboratory for haemoglobin estimation by invasive methods as per the existing protocol being followed at the hospital, and willing to provide informed consent for additional haemoglobin estimation using non-invasive method using ToucHb device (V 3.1.7) were selected.

### Exclusion criteria

Participants with red or yellow conjunctiva (following manufacturer’s guidelines), pregnant women and those who refused to participate with/without providing any reasons were excluded from the study.

### Process of data collection

After obtaining oral informed consent, participants were offered a seat in a comfortable position and asked to gently depress the lower eyelids for appropriate exposure as per the operational manual provided by the manufacturer [20]. We obtained two readings within a span of five minutes. Medical laboratory technician with a diploma in medical laboratory technology collected the data.

Data collector and the research team were trained in the use of the device by a representative of the manufacturer upon our request and supervised in pilot sessions subsequently. The age and sex of the study participants was recorded, and time taken for testing using ToucHb device was recorded for 107 observations. No personal identifying information was collected.

### Laboratory assays

The ToucHb device is a smartphone app based devise that is attached to a phone’s camera and sensors. It captures an image of conjunctiva and uses reflectance photometry to estimate the haemoglobin content in blood in grams per decilitre (g/dL). The detailed method of use is available on the Biosense Technologies Pvt Ltd YouTube channel (https://youtu.be/fe2oneDtkH8?si=5HBTkTx3BiylSjdf) and on the Biosense website [20]. Automated complete blood count (CBC) using the sodium lauryl sulphate method (Sysmex XT-2000i by Sysmex Inc, Kobe, Japan) was used as the reference method.

### Data variables, sources, and tools

Study participants’ age and sex, time took for the ToucHb test, haemoglobin readings from ToucHb and the laboratory assay were entered into epidote [25]. Anaemia was recorded by applying WHO definitions of < 12 g/dL in non-pregnant women, and < 13 g/dL for men [8, 26].

Data analysis was performed in R and OpenEpi version 3.1 (Open-Source Epidemiologic Statistics for Public Health) [27, 28]. Age and time taken to measure ToucHb haemoglobin values was summarised by median and interquartile range (IQR), gender and anaemia was summarised by frequency, sensitivity and specificity and predictive values was performed using a contingency table. Bland-Altman graphs were used to evaluate the agreement between ToucHb and CBC haemoglobin readings. ToucHb device highest value readings were 15 g/dL; for results comparison and for analysis purpose CBC readings of above 15 g/dL were recorded as 15 g/dL.

### Ethics and consent

Study information was provided in the local language (Kannada) and the voice recorded oral consent was obtained from the participants. The study received ethics clearance from the institutional ethics committee of Mysore Medical College and Research Institute and was a sub-study under the THETA project which received ethics clearance from the institutional ethics committee of Institute of Public Health Bengaluru (IPH Bengaluru) (IPH/18-19/E/68). Recorded oral consent is stored in password protected hard disk at IPH Bengaluru office as per the Institutional ethics committee guidelines and will be destroyed after 5 years.

## Results

**Fig 1:**
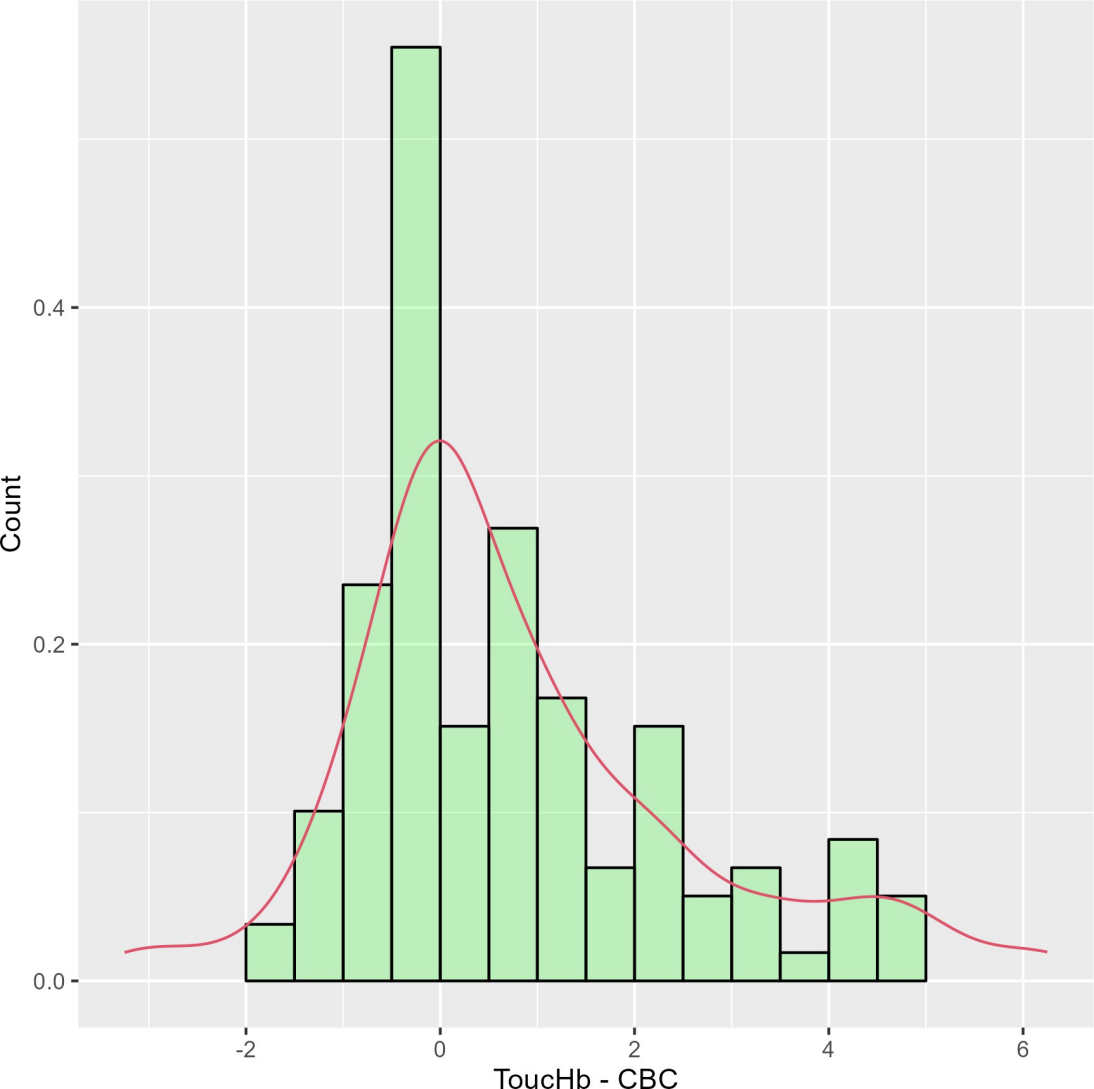
Difference between haemoglobin readings using ToucHb and CBC (reference).

Of the 140 participants who presented at the laboratory during data collection, 127 gave consent to participate in the study. Out of 127 participants 65% (82) identified as female, median age was 37 years (IQR 28–45 years). The prevalence of anaemia among study participants as determined by the ToucHb device was 10.2% (13/127) and from CBC was 28.3% (36/127) (see Table 1). Mean haemoglobin difference between ToucHb and CBC was 1.373 g/dl (ignoring the direction of difference from zero) with limits of agreement ranging from -3.25 to 6.25g/dl. Around 15.7% (20/127) readings of ToucHb haemoglobin had no difference of corresponding CBC readings, while 40.2% (51/127) had less than 1 g/dL, 18.1% (23) differed between 1–2 g/dL, 11% (14/127) differed between 2–3 g/dL and 15% had >3 g/dL. Median time taken to record the haemoglobin value from the ToucHb device was two minutes (IQR 1:59 to 2:59). Differences in haemoglobin readings between ToucHb and CBC showed skewed distribution around zero (Fig 1).

**Table 1:** Prevalence of haemoglobin readings using ToucHb and CBC methods (n = 127).

Severity of anaemia	ToucHb	CBC
Median (IQR)	14.0 (13.25, 14.75)	13.30 (11.70, 15)
Range	8.75–15	5.8–19.4
**Male**		
Anaemic (< 13mg/dL)	1 (2.2%)	3 (6.7%)
Normal HB% (> 13mg/dL)	44 (97.8%)	42 (93.3%)
**Female non pregnant**		
Anaemic (< 12mg/dL)	12 (14.6%)	33 (40.2%)
Normal HB% (> 12mg/dL)	70 (85.4%)	49 (59.8%)

**Fig 2:**
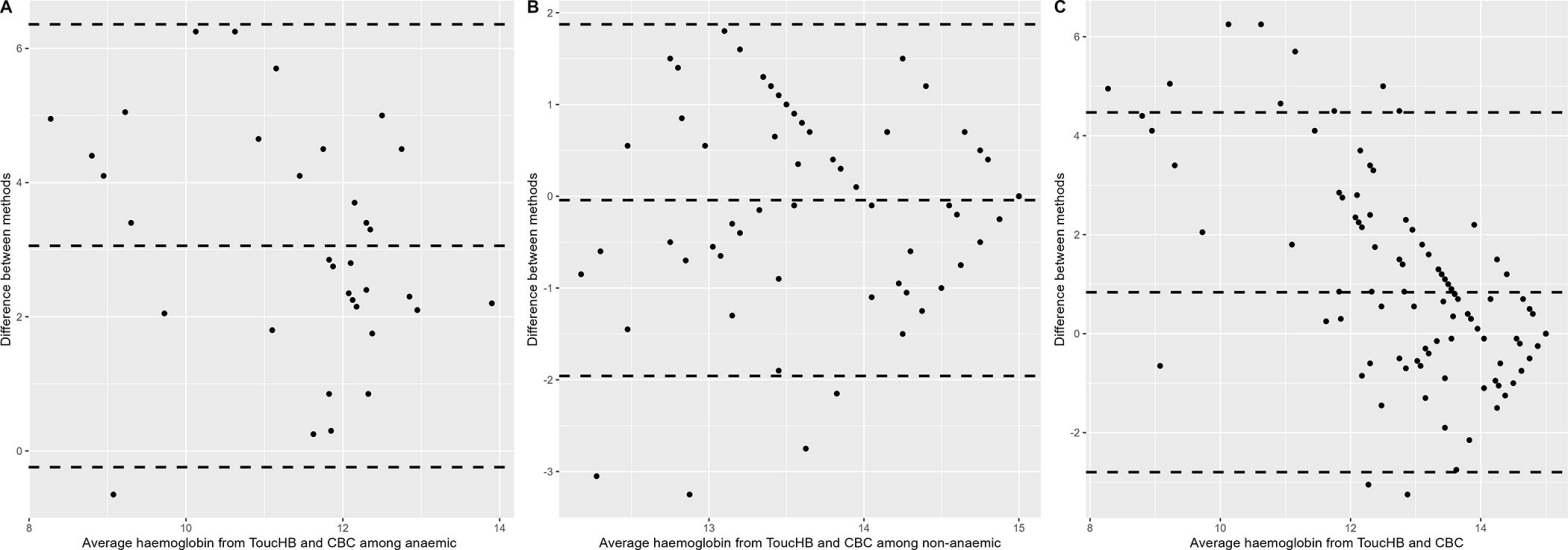
Bland-Altman plot of the haemoglobin using ToucHb and CBC (reference).

The Bland-Altman graph showed mean difference in haemoglobin levels between ToucHb and CBC (Standard method) was 3 g/dL among individuals diagnosed with anaemia with 95% limits of agreement from 6.69 to -0.57 g/dL (Fig 2A). For non-anaemic individuals, the mean difference was -0.04 g/dL with 95% limits of agreement from 3.59 to -3.67 g/dL (Fig 2B). Among all individuals, the mean difference was 0.83 g/dL with 95% limits of agreement from 4.47 to -2.79 g/dL (Fig 2C).

ToucHb shows 22.2% (95%CI 11.72, 38.09) sensitivity, 94.5% (95% CI 87.78, 97.63) specificity, 61.5% (95% CI 35.52, 82.29) positive predictive value (PPV), 75.4% (66.79, 82.43) negative predictive value (NPV), 74.02% (95%CI 65.76, 80.86) diagnostic accuracy, 4.04 (95%CI 1.15, 14.11) likelihood ratio of a positive test and 0.82 (95%CI 0.76, 0.88) likelihood ratio of a negative test corresponding to CBC readings.

## Discussion

In resource limited settings, such as the ones we sought to examine the concertation of haemoglobin among the remote rural population, where diagnostic facility for anaemia is often unavailable or relies on clinical examinations methods like visual assessment of conjunctival pallor, point-of-care devices like ToucHb offer a promising solution. This portable, rapid (2-minute results), and inexpensive device avoids blood draw or a prick, thereby enabling accessible assessments and potentially improving early anaemia detection [18, 20]. While Non-invasive methods like pulse co-oximetry haemoglobin (SpHb) measurement exist in hospital critical care settings with relatively wide levels of agreement with invasive methods [29–32].

Point-of-care devices are more feasible, portable, rapid, and cost-effective for population-based assessments of anaemia in community based household survey and in rural healthcare settings [17]. However, it is essential that these devices are accurate [33]. Inaccurate results can lead to incorrect diagnoses and wrong treatment decisions, which can have serious consequences for research participants, including physical harm, emotional distress, and financial burden. Additionally, they can create ethical dilemmas for healthcare providers and raise concerns about public trust in research.

The ToucHb device is portable, rapid and less expensive than the Hemocue (see S1 Annexure) which is recommended by WHO for surveys to determine haemoglobin estimation at the population level [8]. A study by Sutapa Bandyopadhyay Neogi et al. found that the ToucHB device had a sensitivity of 73.1% and a specificity of 51.5%. However, our study found that the ToucHb had a low sensitivity (22%) and acceptably high specificity for anaemia detection(94%) [21]. This means that it is more likely to correctly identify people who do not have anaemia (true negatives) than it is to correctly identify people who do have anaemia (true positives). The Bland-Altman plot (Fig 2A) shows that the mean difference between the ToucHB readings and the laboratory-measured haemoglobin levels was wider for anaemic patients than for patients with normal haemoglobin levels. This suggests that the ToucHb device may be less accurate for anaemic patients. The estimation of obtaining the ToucHb reading may be affected by various factors, such as excessive ambient light which interfere with the light sensor, peripheral blood circulation to the orbit which can be reduced in anaemic patients, skin pigmentation, and the skin and hydration level, as well as operator technique and experience. In addition, the extent of conjunctiva versus skin must be carefully assessed in various skin complexions, and the device uses artificial intelligence, this algorithm may need to be further trained on a wider range scenario to ensure accurate results for all patients. To ensure valid results, the device may need to be widely tested in diverse participants with multiple skin tones and more iterations of training among diverse population groups are also needed to improve the accuracy of the algorithm.

### Study limitations

Study was conducted among participants who were referred by a physician for testing haemoglobin concentration. Therefore, the findings of this study may not reflect the accuracy of the ToucHb device in the general population. Furthermore, the study duration was limited, and the long-term reliability of the ToucHb device remains unknown.

## Conclusions

Non-invasive devices such as the ToucHb device have potential as a proof of concept, but their application in community health surveys and primary/secondary care settings is limited. The ToucHb device is more appropriate for monitoring established haemoglobin levels than for screening or detecting anaemia in primary/secondary care and community settings. Because of lower accuracy among anaemic individuals, the added value of using this device over clinical examination of pallor is low.

## Supporting information

S1 Code Sample size calculation using R. (RMD)

S1 Annexure Touch HB and HemoCue operational cost comparison for 500 samples. (DOCX)
