# Correction: Validation of ToucHb, a non-invasive haemoglobin estimation: Effective for normal ranges, needs improvement for anaemia detection

**DOI:** 10.1371/journal.pgph.0004137

**Published:** 2024-12-30

**Authors:** Yogish Channa Basappa, Sumanth Mallikarjuna Majgi, Shashidhar Byrappa Hanumanahally, Prashanth Nuggehalli Srinivas

The third author’s name is incorrect. The correct name is Shashidhar Byrappa Hanumanahally. The correct citation is: Channa Basappa Y, Majgi SM, Hanumanahally SB, Srinivas PN (2024) Validation of ToucHb, a non-invasive haemoglobin estimation: Effective for normal ranges, needs improvement for anaemia detection. PLOS Glob Public Health 4(3): e0001541. https://doi.org/10.1371/journal.pgph.0001541
