# Supplementary material for: Validation of ToucHb, a non-invasive haemoglobin estimation: Effective for normal ranges, needs improvement for anaemia detection
Source: PLOS Glob Public Health. 2024 Mar 12;4(3):e0001541. doi: 10.1371/journal.pgph.0001541 (PMC10931486; doi:10.1371/journal.pgph.0001541)
Supplement: S1 Annexure — (DOCX) [file pgph.0001541.s002.docx]

| Device | ToucHB | HemoCue HB 201 |
| --- | --- | --- |
| Purchase cost | Rs 25000 (USD 305 @82)^1^ | Rs 31,000 (USD 378 @82)^2^ |
| Strips | NA | Rs 9450 (USD 115 @82)^3^ |
| Lancets | NA | Rs 595 (USD 7.25 @82)^4^ |
| Alcohol Swab | Rs 449 (USD 5.47 @82)^5^ | Rs 449 (USD 5.47 @82)^5^ |
| Total | Rs 25,449 (USD 310.4 @82) | Rs 41494 (USD 506 @82) |

S1 Annexure: Touch HB and HemoCue operational cost comparison for 500 samples.

Source

1. Biosense. Biosense [Internet]. (ToucHB) Non Invasive Haemoglobin for Anaemia Screening. [cited 2020 Apr 23]. Available from: https://www.biosense.in/touchb.php

2. HemoCue Hb 201 System [Internet]. [cited 2023 Apr 17]. Available from: https://www.indiamart.com/proddetail/hemocue-hb-201-system-19611306888.html

3. Fully Automatic Hemoglobin Hemocue HB 201 Strips [Internet]. [cited 2023 Apr 11]. Available from: https://www.indiamart.com/proddetail/hemocue-hb-201-strips-22090031488.html

4. Recombigen Round Blood Lancet 100 Pieces Glucometer Lancets [Internet]. [cited 2023 Apr 17]. Available from: https://www.amazon.in/Recombigen-Lancet-Pieces-Glucometer-Lancets/dp/B0B5F13HYW/ref=sr_1_6?keywords=lancet&qid=1681729095&sr=8-6

5. Disposable Sterile Antiseptic Cleaning Alcohol Swab, Pack of 500 Swabs [Internet]. [cited 2023 Apr 17]. Available from: https://www.amazon.in/Disposable-Sterile-Antiseptic-Cleaning-Alcohol/dp/B09XMY3QXM/ref=sr_1_7?keywords=alcohol+swabs+for+glucometer&qid=1681729393&sprefix=alcohol%2Caps%2C356&sr=8-7
